# Enhancing cognitive-motor recovery in Rett syndrome: effects of integrated intervention on neuropsychological and motor outcome

**DOI:** 10.3389/fpsyg.2025.1679593

**Published:** 2025-11-05

**Authors:** Rosa Angela Fabio, Martina Semino, Michela Perina

**Affiliations:** ^1^Department of Biomedical, Dental and Morphological and Functional Imaging Sciences, University of Messina, Messina, Italy; ^2^Airett Centre for Research and Innovation, Verona, Italy; ^3^Tice Cooperativa Sociale, Piacenza, Italy

**Keywords:** Rett syndrome, rehabilitation, cognitive-motor rehabilitation, GAIRS scale, intervention, neurodevelopmental disorders

## Abstract

Cognitive-motor integration plays a crucial role in the rehabilitation of individuals with complex disabilities, where dissociated impairments in cognition and movement often hinder global functioning. In this study, we investigated the efficacy of an integrated neurorehabilitation program targeting both neuropsychological and motor domains in patients with Rett Syndrome. Baseline assessments included measures of attention, memory, and temporal sequencing, as well as gross, fine, and graphomotor abilities, evaluated using relevant GAIRS subscales (Global Assessment and Intervention Rating Scale). Nineteen patients were enrolled in an experimental group receiving specialized cognitive-motor training three times a week for two consecutive 5-week periods. A control group of 15 patients participated in standard educational activities without specific cognitive-motor intervention. Performance was evaluated at three time points: T0 (baseline), T1 (after 5 weeks), and T2 (after an additional 5 weeks). Results indicated significant improvements in both neuropsychological and motor functions in the experimental group, with gains observed at both T1 and T2. Notably, a strong and significant correlation emerged between improvements in motor and cognitive measures, underscoring the interdependence of these domains in neurodevelopmental conditions such as Rett Syndrome. These findings support the implementation of integrated cognitive-motor protocols in neurorehabilitation and highlight the value of synchronized interventions to foster global functioning in individuals with complex neurodevelopmental profiles.

## 1 Introduction

Rett syndrome (RTT) is a progressive neurodevelopmental disorder, affecting approximately 1 in 10,000–15,000 females worldwide. The primary cause of classic RTT was identified with *de novo* mutations of the MECP2 gene on the X chromosome (Amir et al., 1999). Subsequent studies have confirmed that MECP2 mutations occur in 90–95% of RTT cases, although rarer variants (e.g., CDKL5, FOXG1) may present with Rett-like features (Bricker and Vaughn, 2024).

RTT typically begins with seemingly normal development until 6–18 months of age, followed by regression in purposeful hand use, verbal skills, and motor abilities. Classical hallmarks include stereotyped hand movements, ataxia, seizures, microcephaly, and breathing irregularities (Downs et al., 2024). The neurodevelopmental regression is linked to loss of MECP2 function, which impairs neuronal maturation, synaptic plasticity, and chromatin regulation (Good et al., 2021).

Children with RTT experience complex disabilities across cognitive, motor, communicative, and autonomic domains. This multifaceted impairment demands rehabilitation programs that address all dimensions, rather than single-domain interventions (Fabio et al., 2025a). A scoping review (Yang et al., 2021) emphasized multimodal therapeutic approaches, from physiotherapy to remote protocols, as more effective in managing functional deficits compared to monotherapies. Although motor therapies, such as treadmill training and environmental enrichment, show promising results, most studies are limited by small sample sizes and heterogeneous methodologies (Yang et al., 2021). Nevertheless, individualized physiotherapy remains vital to maintain gross motor function, prevent contractures, and support daily living (Fonzo et al., 2020; Fabio et al., 2025b; Romano et al., 2020). Recent evidence also suggests the potential for cumulative and sustained benefits of prolonged interventions. Fabio et al. (2025a) reported that a 6-month rehabilitation program led to significant gains in cognitive and communicative abilities in girls with RTT, with improvements maintained at 3-month follow-up.

Given these findings, focusing solely on the motor domain may be insufficient, given the increasingly recognized interdependence between motor and cognitive functions (Young et al., 2022; Kruse Gyldhof et al., 2022). Cognitive-motor integration refers to the interconnected processes that underlie both motor and cognitive functions, wherein the brain’s ability to control movement and process cognitive tasks are not isolated but interdependent (Passaro et al., 2024; Rozensztrauch et al., 2021). Clinical presentation of Rett syndrome in relation to cognitive-motor functioning reveals how impairments in one domain can influence the other. This concept is based on the premise that cognitive and motor functions are supported by interconnected neural systems, including the prefrontal cortex and cerebellum, forming an integrated motor-cognitive network. At the molecular level, MECP2 mutations disrupt several key pathways involved in cognitive-motor integration. Reduced MeCP2 function alters chromatin organization and mRNA/miRNA processing, leading to impaired neuronal maturation and synaptic development, particularly within prefrontal and cerebellar circuits (Good et al., 2021). Notably, MeCP2 deficiency is associated with reduced brain-derived neurotrophic factor (BDNF) expression, which compromises plasticity in the prefrontal-cerebellar pathway and weakens the synergy between motor learning and cognitive regulation. These mechanisms provide a biological rationale for targeting cognitive-motor interaction in RTT rehabilitation. Disruptions in this system, as observed in Rett syndrome, suggest that interventions targeting both domains may offer greater therapeutic benefit (Di Palma et al., 2025). This approach is strategic in neurodevelopmental disorders like Rett syndrome, where deficits in both motor control and cognitive functions co-occur.

Supporting this view, emerging research on cognitive rehabilitation reveals it can modulate neurophysiological activity (e.g., increased beta, decreased delta EEG rhythms), suggesting brain activation in RTT. Transcranial direct current stimulation (tDCS) together with linguistic training improved language abilities and motor coordination in subjects with RTT (Gangemi et al., 2018; Fabio et al., 2020; Fabio et al., 2021; Fabio et al., 2005; Iannizzotto et al., 2020). This supports the intertwined nature of cognitive and motor systems, and the potential for synergistic effects when both are targeted simultaneously.

Delivering such comprehensive care requires the integration of multidisciplinary expertise within specialized environments, where validated assessment tools support personalized and holistic intervention planning (Fonzo et al., 2020). Given the co-occurrence of motor and cognitive impairments in Rett syndrome, a multidisciplinary and integrated approach is essential to maximize functional outcomes. Supporting the feasibility of cognitive training in children with neurodevelopmental disorders, several studies have demonstrated positive outcomes in populations with intellectual disabilities, Down syndrome, and fragile X syndrome (FXS). Children with intellectual disabilities show deficits in both reasoning ability and working memory (WM) that impact everyday functioning and academic achievement. In one study, participants were randomized to a 5-week adaptive WM training program (intervention group) or a non-adaptive version of the program (active control group). Cognitive assessments conducted before, immediately after training, and at 1-year follow-up revealed that training progress predicted transfer to WM and comprehension of instructions, with higher progress associated with greater improvements (Söderqvist et al., 2012). Similarly, a study in children with Down syndrome evaluated the impact of a computerized visuospatial memory training intervention delivered in schools over a 10–16 week period, with teaching assistants supporting individualized sessions. Twenty-one children were randomly allocated to either the intervention or a waiting-list control group. Children in the intervention group demonstrated significant improvements in both trained and non-trained visuospatial short-term memory tasks, and these gains were sustained 4 months later. These findings indicate that computerized cognitive training in a school setting is not only feasible but also effective for children with Down syndrome (Bennett et al., 2013). In addition, individuals with fragile X syndrome (FXS) a condition that, like RTT, presents with synaptic dysfunction and overlapping cognitive-motor impairment and that is often present with deficits in working memory (WM) and other executive functions. Preliminary findings evaluating the Cogmed JM program, a computer-based WM intervention, demonstrated that WM training is feasible in the FXS population. In that study 8 participants completed the 5-week training. Baseline characteristics, training progress, and parental impressions were analyzed, showing that the program could be successfully implemented, although a certain baseline level of cognitive ability was required (Au et al., 2014). These results support the notion that structured, computer-assisted cognitive training is practicable in populations with neurodevelopmental disorders and can provide a foundation for integrated cognitive-motor rehabilitation strategies. This is particularly relevant because studies have shown that motor learning is tightly linked to cognitive functions such as attention, executive processing, and memory, which are essential for acquiring, retaining, and transferring new motor skills (Rajda et al., 2025). For example, in moderate-to-severe traumatic brain injury (TBI), rehabilitation outcomes significantly improve when motor and cognitive interventions are delivered in a coordinated manner (Shen et al., 2025). Dual-task training and neurogaming platforms are increasingly used in TBI to stimulate both motor and cognitive systems simultaneously, demonstrating enhanced engagement and recovery. Similarly, recent meta-analytic evidence in autism spectrum disorder (ASD) confirms that physical activity interventions produce medium to large improvements in executive functions such as inhibitory control, cognitive flexibility, and working memory (Hou et al., 2024; Voniati et al., 2025; Wandin et al., 2023). Notably, interventions that require dynamic movement coordination and attentional engagement showed the strongest effects, further supporting the synergy between motor and cognitive systems (Hirano et al., 2023; Tost et al., 2025). Consistent results were also observed in children and adolescents with attention-deficit/hyperactivity disorder (ADHD). A recent systematic review and meta-analysis by Fang et al. (2024) demonstrated that cognitively combined or cognitively engaging physical activity interventions significantly improved executive function, motor competence, and reduced core ADHD symptoms. Regarding the same disorder, Zhao et al. (2024) applied an integrated cognitive and physical activity intervention to a sample of 90 children with ADHD. After 4 weeks of intervention (three sessions per week), a reduction in disease severity was observed, further supporting the role of integrated programs promising non-pharmacological interventions for ADHD. Extending this perspective, Wang et al. (2015) conducted a meta-analysis showing that motor-based interventions, especially those incorporating cognitive demands, such as dual-task paradigms, yielded significantly greater improvements in executive functions like inhibition, attention, and working memory. These findings highlight the unique advantage of cognitively engaging physical activity, where the simultaneous activation of motor and cognitive systems appears to drive stronger therapeutic effects. These results also provide a direct rationale for RTT: given the characteristic stereotyped movements and poor coordination, embedding structured and externally guided motor tasks within a cognitively engaging framework (e.g., adaptive progression and computer-assisted training) may help optimize residual neuroplasticity, drawing on validated principles from related neurodevelopmental disorders.

Furthermore, even in healthy older adults, structured cognitive-motor training paradigms, such as combining balance tasks with simultaneous cognitive challenges, have been shown to enhance postural control and gait stability together with cognitive abilities, compared to single-task conditions (Li et al., 2024). In line with this, Gao et al. (2024) outlined in their evidence-based review focusing on children with developmental coordination disorder, that interventions targeting motor deficits alone are insufficient to improve daily functioning. Instead, they advocate for a multidisciplinary model combining physical, cognitive, perceptual, and environmental strategies, including dual-task training and motor imagery, to enable children to acquire problem-solving skills, enhance their motor planning and execution abilities. Consistently, Ferrari et al. (2023) demonstrated in a large sample of children with intellectual and developmental disability that visuospatial and sensorimotor abilities are critical predictors of social cognitive functions such as emotion recognition and theory of mind. This evidence highlights that targeting motor and spatial skills may have downstream effects on higher-order cognitive and social domains, further strengthening the rationale for integrated approaches in congenital neurodevelopmental disorders. Taken together, these findings strongly support the need for holistic, integrated rehabilitation strategies. Considering the cognitive–motor interdependence in Rett syndrome, similar approaches may offer particularly meaningful benefits, enhancing not only isolated functional domains but also their dynamic interaction.

Although existing RTT rehabilitation studies are often limited by small sample sizes and methodological heterogeneity, the present study addresses these issues through design optimization. Outcomes were assessed using RTT-validated standardized tools (GAIRS and RARS), and repeated evaluations were conducted at multiple time points, including a 3-month follow-up. These features enhance the reliability and interpretability of the findings, allowing for a more robust evaluation of intervention effects despite the relatively small sample size.

### 1.1 Hypotheses

Based on existing theoretical and empirical evidence highlighting the interdependence between cognitive and motor domains in both typical and atypical development, this study aims to assess the efficacy of an integrated rehabilitation program in individuals with Rett Syndrome. Specifically, the following hypotheses are proposed:

*Hypothesis 1 (H1):* Patients receiving the integrated intervention will show greater improvements in neuropsychological functions (attention, memory, temporal sequencing) compared to controls who participate in standard educational activities only.

*Hypothesis 2 (H2):* Patients in the experimental group will exhibit greater improvements in motor abilities (gross, fine, and graphomotor skills) compared to controls.

*Hypothesis 3 (H3):* The cognitive and motor gains observed in the experimental group will be maintained or further enhanced after the second treatment cycle, indicating a cumulative effect of the intervention.

*Hypothesis 4 (H4):* Within the experimental group, a positive correlation will emerge between improvements in cognitive and motor domains, supporting the notion of functional integration between these systems.

## 2 Materials and methods

### 2.1 Participants

A total of 34 girls with RTT took part in the study. Participants were randomly assigned to two groups: an Experimental Group (*n* = 19; *M*_age_ = 11.54 years (SD = 3.23); range 4–17) and a Control Group (*n* = 15; *M* = 11.46 years (SD = 4.01); range 4–17).

Inclusion criteria were a confirmed diagnosis of RTT (with documented MECP2 or FOXG1 mutation) regular attendance at a rehabilitative center or clinic, availability and commitment of the center to participate for the full duration of the study, and informed consent signed by the families after being fully informed about the nature and structure of the intervention.

At recruitment, each participant was assessed using the Rett Assessment Rating Scale (RARS) and the Vineland Adaptive Behavior Scale (VABS). Demographic and clinical data were also collected to verify inclusion criteria. The experimental and the control groups were comparable at baseline. Specifically, no significant differences were found between the Experimental Group (M_RARS = 63.43, SD = 6.71; M_VABS = 93.68, SD = 23.13) and the Control Group (M_RARS = 63.43, SD = 9.81; M_VABS = 94.60, SD = 32.55) on either the Rett Assessment Rating Scale (RARS) or the Vineland Adaptive Behavior Scale (VABS) (all *p*s > 0.05) (Table 1).

**Table 1:** Descriptive statistics of participants.

ID	Age	Mutation	RARS	VABS
Experimental group				
1	4	Mecp2 T158M	66	75
2	10	Mecp2 T158M	75.5	75
3	4	MECP2 R 306C	46	100
4	21	Mecp2 Arg168	58.34	84
5	17	Mecp2	71	71
6	17	Mecp2 C.952	69.5	109
7	16	Mecp2 T158M	64	136
8	16	MECP2 c.763C > T p. Arg255X	62	91
9	13	MECP2 R255X	64	111
10	6	MECP2 c.808C > T (p. R270X)	65.5	104
11	14	MECP2 P133C	72	151
12	9	MeCP2	65	110
13	12	MeCP2	60.5	108
14	4	MeCP2 P152R	63.5	74
15	16	MECP2 C.316	64.5	84
16	11	MeCP2	67.5	78
17	7	MECP2	54	70
18	10	MECP2	66.5	69
19	6	MECP2 C965C	58	80
Control group				
20	7	Mecp2	66	71
21	10	Mecp2	80	150
22	4	Mecp2	56	75
23	17	Mecp2	75	149
24	17	Mecp2	73.5	152
25	17	Mecp2	65	69
26	16	Mecp2	59	91
27	16	Mecp2	68.5	95
28	13	Mcp2 c.806	43	65
29	6	Mecp2	57	69
30	14	Mecp2	71	120
31	9	Mecp2 T158M	63	100
32	12	Mecp2	64	70
33	4	Mecp2	49	68
34	7	Mecp2 R294x	61.5	75

### 2.2 Study design

This study followed a longitudinal, controlled experimental design composed of three phases: a baseline assessment (T0), a midpoint evaluation after 5 weeks of intervention (T1), and a post-intervention evaluation after an additional 5 weeks (T2).

After recruitment and informed consent, participants were randomly assigned to one of two groups: an experimental group, which received a structured cognitive rehabilitation program, or a control group, which followed standard in-person educational activities. Simple random sampling was used: the allocation sequence was generated by an independent research assistant using a computer-generated random number table and sealed in opaque envelopes. Envelopes were only opened after participants completed the baseline assessment to ensure allocation concealment. All outcome evaluations were performed by certified therapists specialized in RTT, who were blinded to group allocation to minimize assessment bias. Schoolteachers were aware of group assignments due to the nature of the intervention, but were instructed not to disclose group information to the evaluators.

The standardized tools used during baseline assessment included the Rett Assessment Rating Scale (RARS) Fabio et al. (2025b) to evaluate syndrome severity and the Vineland Adaptive Behavior Scales (VABSs) (Sparrow et al., 2016) to assess adaptive functioning.

The primary measure was the Global Assessment and Intervention Rating Scale (GAIRS) (Fabio et al., 2021; Caprì et al., 2020) a multidimensional checklist tailored for individuals with Rett Syndrome and complex neurodevelopmental profiles. Only three GAIRS subscales (i.e., Neuropsychological Concepts, Hand motor skills and Global motor abilities) were analyzed in this study.

### 2.3 Assessment and measures

Prior to the intervention, information about the participants’ characteristics was gathered using the RARS and VABSs.

RARS is a validated clinical tool used to evaluate the severity of RTT symptoms across seven functional domains: cognitive, sensory, motor, emotional, autonomy, typical characteristics, and behavior. The scale includes 31 items rated on a 4-point Likert scale (1 = within normal limits; 4 = marked abnormality), allowing classification of severity into mild (0–55), moderate (56–81), or severe (>81). The instrument demonstrates high psychometric properties, including high internal consistency (Cronbach’s alpha = 0.912).

The VABSs assess four core domains of adaptive functioning: communication, daily living skills, socialization, and motor abilities. Each item is rated by an interviewer (2 = always, 1 = sometimes, 0 = rarely/never), and domain scores are summed to yield an overall adaptive behavior composite. VABSs have well-established reliability indices across domains, with split-half reliability ranging from 0.70 to 0.95 and interrater reliability from 0.62 to 0.75.

The GAIRS Checklist was administered at three time points (T0, T1, and T2) and served as the primary outcome measure for evaluating participants’ cognitive and communicative functioning over time.

To ensure scoring objectivity, two GAIRS-trained therapists independently rated 20% of the participants’ assessment data. Inter-rater reliability was assessed using intra class correlation coefficients (ICC), yielding ICC = 0.89 for the Neuropsychological Concepts subscale, ICC = 0.91 for the Hand Motor Skills subscale, and ICC = 0.87 for the Global Motor Abilities subscale, all indicating good reliability (ICC > 0.8).

GAIRS encompasses 10 functional domains: basic prerequisites, neuropsychological skills, basic cognitive concepts, advanced cognitive concepts, communication, emotional affective abilities, hand motor skills, graphomotor abilities, gross motor abilities, and autonomy in daily life. In this study, the intervention specifically targeted two subscales: the Basic Cognitive Concepts area (e.g., object recognition, form and color discrimination, spatial and temporal understanding, and cause–effect relationships) and the Communication.

Abilities area (e.g., comprehension and expression through gestures, images, and verbal output). The items included in the targeted subscales are briefly described in Table 2, while the evaluation procedures and examples of the materials used during the assessment are provided in Appendix A. Recent studies have demonstrated that GAIRS is a reliable and comprehensive instrument for evaluating individuals with Rett syndrome in both traditional and remote settings (Fabio et al., 2022).

**Table 2:** Contents of the three subscales: neuropsychological concepts, hand motor skills and global motor abilities.

Ability	Description
Neuropsychological concepts	
Temporal orientation	The ability to understand and place events in time (e.g., today, yesterday, tomorrow).
Spatial orientation	The ability to recognize and use spatial concepts such as above, below, in front, and behind.
Memory span	The ability to actively retain a limited amount of information for a short period of time.
Logical sequencing:	The ability to arrange information or events in a logical and coherent order.
Categorization	The ability to group objects or concepts based on shared characteristics or specific criteria.
Temporal concepts	The understanding of more complex temporal notions, such as days of the week, seasons, and time of day.
Hand motor skills	
Eye–hand coordination	The ability to coordinate visual input with hand movements to perform tasks such as reaching or manipulating objects.
Lateralization	The preference or dominance in using one side of the body (e.g., right or left hand, foot, or eye) for specific tasks.
Approach movement	The ability to initiate and direct movement toward an object or target in space.
Touch	The intentional movement of the hand or fingers to establish contact with an object as part of an action or task.
Grasping	The ability to effectively close the hand around an object in order to hold or manipulate it.
Release movement	The controlled ability to let go of an object by opening the hand or relaxing grip appropriately.
Placement movement	The ability to position or place an object in a precise or intentional location using the hands.
Bimanual coordination	The ability to use both hands together in a coordinated and purposeful way to complete tasks.
Pushing or pulling	The ability to apply force to objects in order to move them either away from or toward the body.
Global motor abilities	
Static balance	The ability to maintain a stable and aligned body position while standing.
Sitting posture	The ability to maintain a correct and balanced seated position.
Parachute reactions	Protective extension movements of the arms used to prevent falls when balance is lost.
Rolling: supine to side	The ability to roll the body from lying on the back to lying on the side.
Rolling: supine to prone	The ability to roll the body from lying on the back to lying face down.
Supine to sitting on floor	The ability to move from lying on the back to sitting up on the floor independently.
Sitting to standing on floor	The ability to rise from a seated position on the floor to a standing position.
Sitting to standing on chair	The ability to transition from sitting on a chair to standing upright.
Standing to sitting on floor	The ability to lower oneself from standing to sitting on the floor in a controlled manner.
Standing to sitting on chair	The ability to sit down on a chair from a standing position in a coordinated way.
Walking	The ability to move forward with alternating steps while maintaining balance and coordination.
Spatial orientation (standing)	The ability to understand and respond to spatial relationships with the body in an upright position.
Obstacle crossing	The ability to step over or around objects in the walking path without losing balance.
Running	The ability to move quickly on foot with both feet off the ground during the stride phase.
Stairs up and down	The ability to ascend and descend stairs using coordinated leg movements and balance.
Jumping	The ability to push off the ground with both feet to lift the body into the air and land safely.
Dynamic balance	The ability to maintain stability while the body is in motion, such as during walking, turning, or transitioning between positions.
Playing with ball	The ability to interact with a ball through actions like throwing, catching, or kicking, requiring coordination.
Inclined surface walking	The ability to walk up or down sloped surfaces while maintaining stability and control.

For the experimental group, during the sessions, the therapist also monitored several additional parameters to better understand the participants’ engagement and cognitive functioning. One key aspect observed was sustained attention on the general activity, referring to the child’s ability to remain focused on the proposed tasks.

The therapist also recorded how often attention prompts or physical support were needed within 10-minute intervals, offering insight into the level of support each child required to maintain participation.

Another parameter was the number of correct choices made during an activity session, reflecting the child’s ability to interact meaningfully with the proposed tasks through tasks presented on a computer screen with the support of an eye-tracker. Lastly, the average time spent exploring the visual field before selecting a response was considered, providing information about the participant’s approach to processing and responding within the communicative setting.

It should be noted that these additional evaluations were performed through direct observation of the child’s behavior during the training activities described in the Procedure section, and not through separate cognitive tests. The eye-tracker was used solely as a visual support to reinforce the cognitive exercises, and its recordings were not analyzed or reported as study outcomes.

### 2.4 Procedure

After initial recruitment and consent, all participants underwent a baseline assessment to determine eligibility and establish initial functional profiles. This included administration of the Rett Assessment Rating Scale (RARS) to evaluate syndrome severity, the Vineland Adaptive Behavior Scales (VABSs) to assess adaptive functioning, and the Global Assessment and Intervention Rating Scale (GAIRS) to capture cognitive and communicative abilities across multiple domains.

Participants were then randomly assigned to the experimental or control group. The experimental group received 5 weeks neuropsychological and motor rehabilitation program, conducted at school by their therapist. The intervention targeted selected items from the GAIRS subscales for neuropsychological concepts, hand motor skills and global motor abilities. To ensure engagement and maximize educational collaboration, the intervention consisted of at least three sessions per week, each lasting approximately 1 h, although session duration was adapted to each participant’s attention span. Sessions were conducted during school hours, in quiet, structured environments within the school setting. The 10 weeks intervention comprised approximately 62 sessions (around 62 h of training) per participant, although the exact number could vary slightly due to factors such as illness or school holidays.

When possible, activities included peer involvement, encouraging participation in simple shared tasks to support communication and social interaction.

All necessary cognitive enhancement materials and communication tools were provided to the school and adapted to the developmental level and functional profile of each participant. These included visual support, representing symbolic communication systems designed to support attention, comprehension, and expressive abilities.

Each session followed a consistent structure with the following activities:

1. Warm-up activities (5–10 min): Designed to engage attention and prepare children for structured tasks. Examples included singing simple songs, following visual cues or performing assisted arm movements.
2. Cognitive and neuropsychological skills enhancement exercises (20–40 min): Targeted temporal orientation, spatial orientation, memory span (small sets of objects), simple categorization, and logical sequencing using picture cards. Tasks were adapted to each child’s developmental level.
3. Fine motor and gross motor activities (20–40 min): Fine motor tasks included grasping, release, touch, eye–hand coordination, and bimanual coordination using objects such as stacking cups or placing items in containers. Gross motor activities involved sitting posture, static balance, rolling supine to side, supine to sitting, obstacle crossing, assisted walking, and playing with a ball.

Sessions were consistently structured to maintain predictability, enhance engagement, and support skill generalization.

The quantitative parameters of each activity were individualized on the basis of each child’s baseline GAIRS assessment. Task difficulty was progressively increased after the child achieved at least three consecutive sessions with predominantly correct responses, ensuring both adaptability and gradual skill acquisition.

A detailed summary of the intervention protocol, including task examples, time allocation, and progression criteria, is provided in Appendix B.

**Figure 1:**
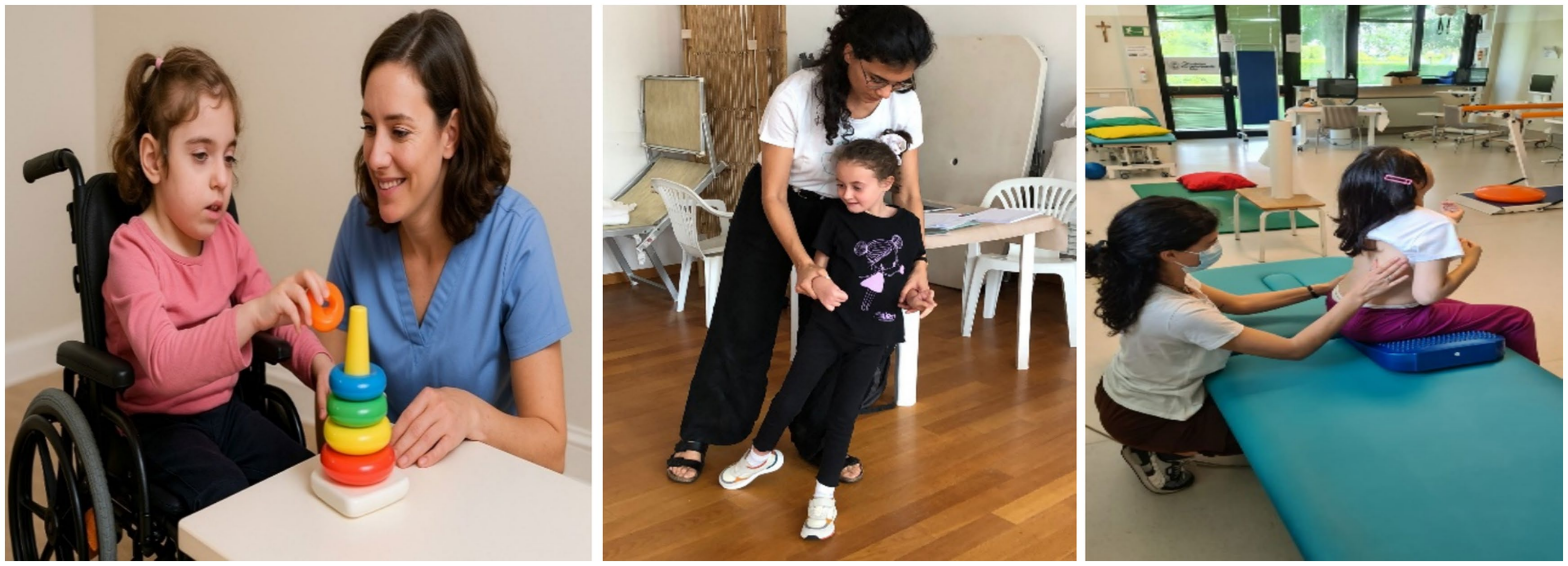
Examples of rehabilitation activities conducted during the intervention. The images show a child with Rett syndrome supported by therapists and educators while performing different tasks: a fine motor exercise involving stacking colored rings, assisted walking for gross motor training, and postural control activity on a therapy table.

Figure 1 depicts the therapist and the child actively engaged in a fine motor exercise and two gross motor activities.

**Figure 2:**
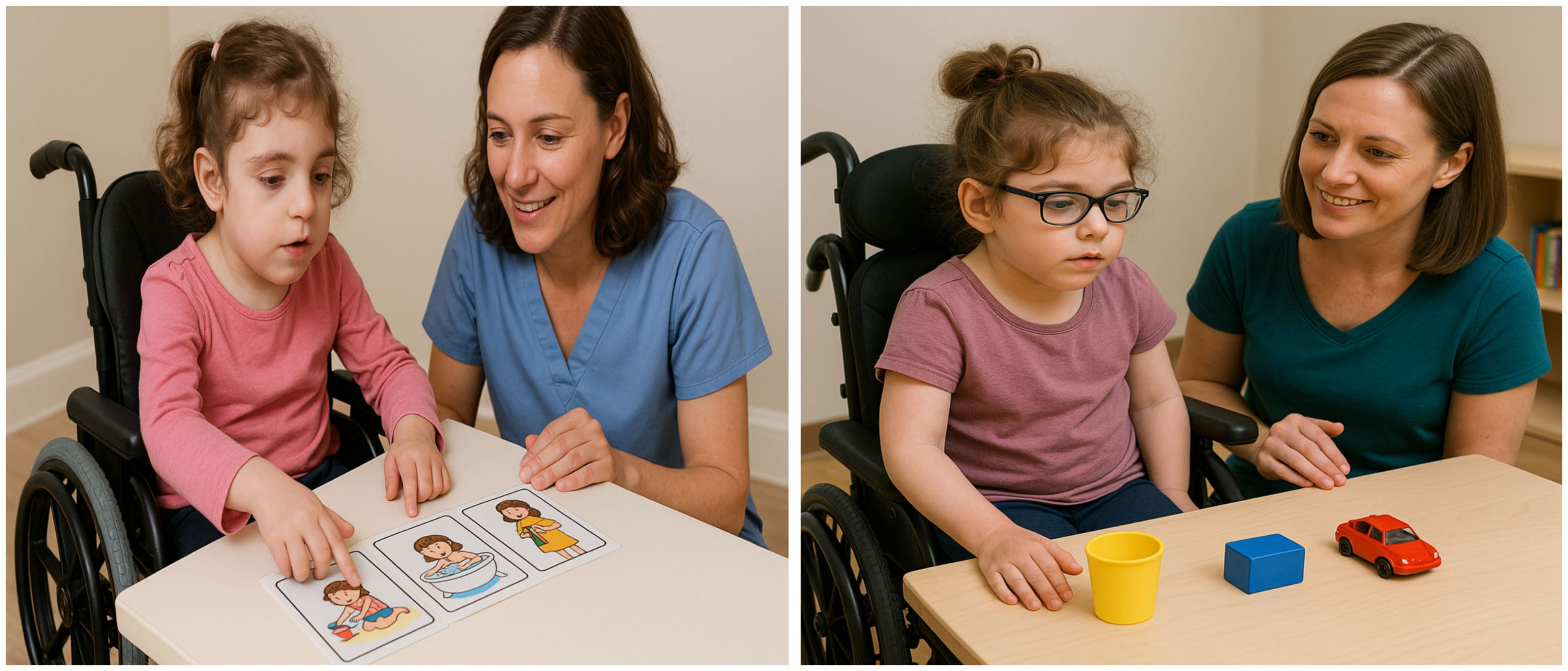
Examples of neuropsychological tasks carried out during the intervention. The images show two girls with Rett syndrome engaged in different activities with a therapist: in the first, the child is identifying the correct temporal sequence of a short, illustrated story; in the second, the child is involved in a memory task using simple objects placed on the table.

Figure 2 illustrates examples of neuropsychological tasks conducted during the intervention. The control group followed the standard educational program.

Both groups were evaluated at the following time points:

- T0 (Baseline): Prior to intervention
- T1 (Post-test 1): After 5 weeks of training
- T2 (Post-test 2): After 10 weeks of training

**Figure 3:**
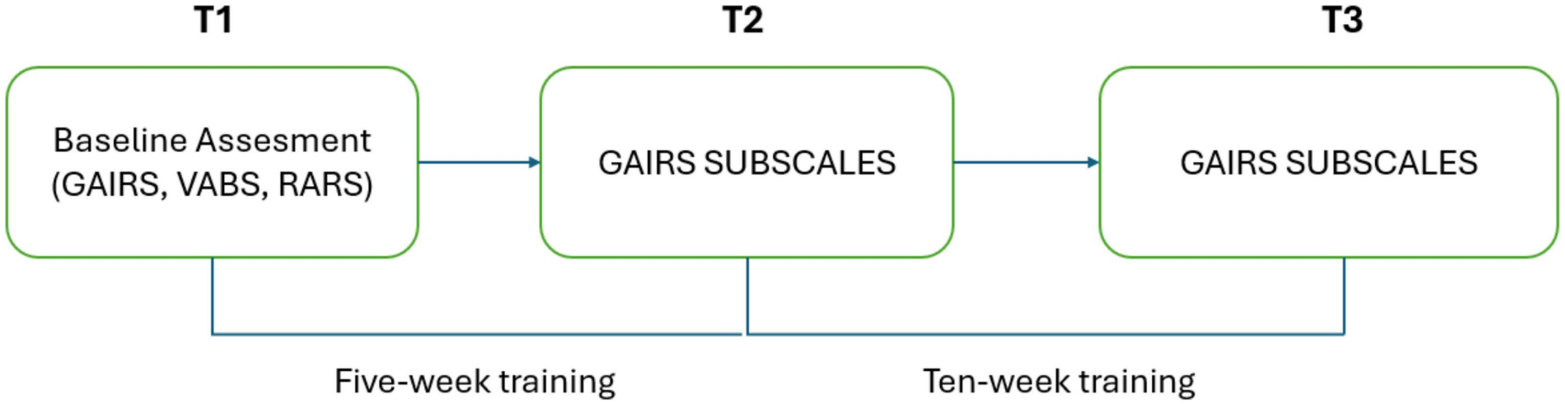
Graphical timeline of the study procedure.

At each point, the previously mentioned subscales of the GAIRS Checklist were administered as the primary outcome measures to monitor changes in cognitive and communicative functioning. For the experimental group, additional measures were collected by the therapist, including sustained attention to the general activity, the number of attention prompts and physical supports required within a 10-min period, the number of correct choices made within 1 h, and the mean exploration time before providing an answer. A graphical representation of the procedure can be found in Figure 3.

### 2.5 Statistical analysis

Statistical analyses were performed using IBM SPSS Statistics (Version 24; IBM Corp., Armonk, NY, USA).

Data from the three subscales of the GAIRS Checklist were analyzed following standardized procedures (Fabio et al., 2022) and an average score was calculated for each subscale. Scores ranged from 1 to 5, with higher scores indicating greater performance within the corresponding functional domain.

In addition to the GAIRS subscales, four observational indicators (sustained attention, attention prompts, number of correct choices, and mean exploration time) were collected during the intervention to provide complementary behavioral information.

These indicators are not part of the standardized GAIRS Checklist and were therefore excluded from the ANOVA models. They were reported for descriptive purposes only, to qualitatively illustrate behavioral changes accompanying standardized functional improvements.

The normality of the data distribution was assessed using both the Shapiro–Wilk test and visual inspection; all *p*-values exceeded 0.05, confirming normality at all three assessment points (T0, T1, T2).

Three separate mixed-design ANOVAs were then conducted—one for each functional domain.

1. Neuropsychological skills: 2 (Groups: Experimental, Control) × 7 (Functions) × 3 (Time points: T0, T1, T2)
2. Fine motor abilities: 2 × 10 × 3
3. Gross motor abilities: 2 × 19 × 3

Mauchly’s test was used to assess the sphericity assumption for all repeated-measures factors (Time and Functions). If the assumption of sphericity was violated (*p* < 0.05), the Greenhouse–Geisser correction was applied to adjust the degrees of freedom. All reported *F*-values, *p*-values, and partial eta squared (η_p_^2^) reflect the corrected values when appropriate.

In addition, for each domain, a separate 2 (Group) × 3 (Time) mixed ANOVA was conducted on the composite score, calculated as the mean of standardized subscale scores, to assess global functional improvement.

Pairwise comparisons among time points were conducted to interpret significant main or lower-order interaction effects (e.g., Time × Group), rather than to probe non-significant higher-order interactions. Bonferroni correction was applied to adjust for multiple comparisons. When significant effects emerged, effect sizes were calculated to quantify their magnitude. For the ANOVA results, partial eta squared (η_p_^2^) was used as a measure of effect size and categorized according to established guidelines (Cohen, 1988). Additionally, paired-samples *t*-tests comparing performance across different time points were accompanied by Cohen’s *d* effect sizes, interpreted as small (0.2), medium (0.5), or large (0.8) changes. These effect sizes provided further insight by illustrating the extent of change between specific measurement occasions, complementing the overall ANOVA findings.

Finally, an exploratory correlation analysis was conducted to examine whether improvements in cognitive and motor domains were associated within the experimental group. For each participant, delta scores (Δ) were computed by subtracting baseline (T0) performance from post-intervention (T2) performance on the composite indices of cognitive and motor functioning. Thus, higher Δ values indicated greater improvement over time.

This exploratory analysis was performed only for the experimental group, as this was the only condition that received the combined cognitive–motor training and therefore could show covariation between changes across domains. Pearson’s correlations were used, and the results were interpreted descriptively given the limited sample size and exploratory nature of the analysis.

## 3 Results

### 3.1 Neuropsychological functions

Table 3 presents the descriptive statistics (means and standard deviations) for each neuropsychological skill across the three time points (T0, T1, T2) in the experimental and control groups. A 3-way mixed ANOVA with Time (T0, T1, T2) as within-subjects factor and Group (Experimental, Control) and Function as between-subjects factors revealed a significant main effect of Time, *F*(2, 116) = 15.72, *p* < 0.001, *η*^2^ = 0.21, indicating overall improvement across assessments. The main effect of Group was not significant, *F*(1, 58) = 2.13, *p* = 0.15, *η*^2^ = 0.04, suggesting that the two groups did not differ in their overall performance. A significant main effect of Function emerged, *F*(7, 406) = 5.84, *p* < 0.001, *η*^2^ = 0.09, indicating variability across the different neuropsychological measures. The interaction between Time and Group was significant, *F*(2, 116) = 6.52, *p* = 0.002, *η*^2^ = 0.10, showing that the groups followed different improvement trajectories over time. The Time × Function interaction was also significant, *F*(14, 812) = 3.27, *p* < 0.001, *η*^2^ = 0.05, whereas both the Group × Function interaction, *F*(7, 406) = 1.02, *p* = 0.42, *η*^2^ = 0.02, and the three-way Time × Group × Function interaction, *F*(14, 812) = 1.56, *p* = 0.09, *η*^2^ = 0.03, were not significant. These findings support Hypothesis 1 (H1), which predicted greater cognitive improvements in the experimental group.

**Table 3:** Descriptive statistics of neuropsychological subscales at T0, T1, and T2 for experimental and control groups.

Cognitive skill	Group	T0	T1	T2
Sustained attention on general activity(s)	Experimental	6.25 (4.20)	12.88 (6.24)*	18.25 (7.01)**
	Control	7.20 (7.33)	11.20 (6.27)	12.60 (6.97)
Attention prompts and physical support (number of supports, 10 min)	Experimental	21.88 (3.72)	14.38 (2.93)**	12.63 (5.24)**
	Control	21.33 (4.94)	21.67 (5.23)	21.27 (4.94)
Number of correct choices in 1 h	Experimental	1.25 (1.04)	3.63 (0.92)**	4.38 (1.06)**
	Control	1.87 (1.25)	2.27 (1.44)	2.27 (1.44)
Mean exploration time before answering	Experimental	14.25 (5.70)	7.38 (3.50)**	4.00 (4.47)**
	Control	13.87 (6.08)	13.33 (4.78)	13.20 (4.81)
GAIRS neuropsychological subscales				
Temporal orientation	Experimental	1.37 (0.43)	1.78 (0.45)	2.22 (0.65)**
	Control	1.60 (0.91)	1.87 (1.06)	2.07 (1.16)
Spatial orientation	Experimental	1.00 (0.00)	1.00 (0.00)	1.38 (0.74)
	Control	1.53 (1.06)	1.60 (1.06)	1.80 (1.15)
Memory span	Experimental	1.00 (0.00)	1.70 (0.40)	1.60 (0.30)
	Control	1.40 (0.51)	1.60 (0.74)	1.87 (0.92)
Logical sequencing	Experimental	1.00 (0.00)	1.20 (0.10)	1.20 (0.10)
	Control	1.13 (0.35)	1.20 (0.56)	1.33 (0.72)
Categorization	Experimental	1.00 (0.00)	1.70 (0.36)*	2.23 (0.44)**
	Control	1.07 (0.26)	1.07 (0.26)	1.07 (0.26)
Temporal concepts	Experimental	1.00 (0.00)	1.13 (0.35)	1.13 (0.35)
	Control	1.07 (0.26)	1.07 (0.26)	1.07 (0.26)
Mean composite score for neuropsychological skills	Experimental	1.57 (0.33)	2.01 (0.20)	2.19 (0.27)
	Control	1.79 (0.34)	1.88 (0.41)	1.92 (0.39)

Values are means with standard deviations in parentheses. The experimental group (*n* = 19) and control group (*n* = 15) were assessed at three time points: T0 (baseline), T1, and T2. *indicate significant difference from T0: *p < 0.05; **p < 0.01.

The four additional indicators (sustained attention, attention prompts, number of correct choices, and mean exploration time) were observational measures routinely collected during the intervention but not part of the standardized GAIRS Checklist. These data were reported for descriptive purposes only, to qualitatively illustrate behavioral patterns accompanying functional improvements. They were not included in the ANOVA models, as they differ in measurement scale, directionality, and psychometric validation from the standardized subscales.

Pairwise comparisons among time points indicated that participants in the experimental group significantly improved in sustained attention from T0 to T1 (*p* < 0.05, *d* = 0.92) and from T0 to T2 (*p* < 0.01, *d* = 1.15), with mean times increasing from 6.25 (SD = 4.20) seconds at baseline to 18.25 (SD = 7.01) seconds at follow-up. The control group showed only modest gains. Although the control group showed a slight increase in sustained attention from T0 (*M* = 7.20, SD = 7.33) to T2 (*M* = 12.60, SD = 6.97), this change was not statistically significant, *t*(29) = 1.61, *p* = 0.12, *d* = 0.29. Such a modest improvement may reflect normal developmental or practice-related effects associated with standard educational activities (e.g., regular classroom engagement), rather than specific intervention effects. The magnitude of increase was substantially lower than that observed in the experimental group (Δ = +12.0 s vs. +5.4 s), supporting the selective impact of the training program. Attention prompts and physical supports decreased markedly in the experimental group (from 21.88, *SD* = 3.72 at T0 to 12.63, *SD* = 6.24 at T2, *p* < 0.01, *d* = 1.12), while remaining stable in the control group.

Problem-solving skills also improved significantly in the experimental group: the number of correct choices increased from 1.25 (*SD* = 1.04) at T0 to 4.38 (*SD* = 1.06) at T2 (*p* < 0.01, *d* = 1.10), and the time spent exploring before answering decreased from 14.25 (*SD* = 5.70) seconds to 4.00 (*SD* = 4.47) seconds (*p* < 0.01, *d* = 0.98). No substantial changes were observed in the control group. Moreover, temporal orientation and categorization abilities significantly increased in the experimental group from T0 to T2 (*p* < 0.01, *d* = 1.23), while spatial orientation and temporal concepts did not show meaningful changes in either group.

Overall, the mean composite score for neuropsychological skills increased from 1.57 (SD = 0.33) at T0 to 2.19 (SD = 0.27) at T2 in the experimental group, reflecting global cognitive improvement. In contrast, the control group showed only marginal gains. These patterns support H1 and are consistent with H3, which anticipated cumulative improvements after the second treatment cycle.

### 3.2 Fine motor functions

Table 4 summarizes the scores for fine motor skills. The ANOVA conducted on fine motor functions showed a significant main effect of Time, *F*(2, 116) = 9.34, *p* < 0.001, *η*^2^ = 0.14, confirming performance gains across sessions. The main effect of Group was not significant, *F*(1, 58) = 0.83, *p* = 0.37, *η*^2^ = 0.01, and a significant main effect of Function emerged, *F*(6, 348) = 4.11, *p* < 0.001, *η*^2^ = 0.07. The Time × Group interaction reached significance, *F*(2, 116) = 4.52, *p* = 0.013, *η*^2^ = 0.07, indicating greater improvement in the Experimental group compared with the Control group. The Time × Function interaction was significant, *F*(12, 696) = 2.78, *p* = 0.002, *η*^2^ = 0.05, whereas the Group × Function, *F*(6, 348) = 1.27, *p* = 0.27, *η*^2^ = 0.02, and the three-way Time × Group × Function interactions, *F*(12, 696) = 1.18, *p* = 0.29, *η*^2^ = 0.02, were not significant. Although the three-way Time × Group × Function interaction was not significant, the two-way Time × Group interaction for the fine motor domain was significant, indicating differential change between groups over time. To interpret this significant two-way effect, we conducted planned simple-effects analyses, examining within-group pairwise comparisons across time points for each fine-motor subscale. These follow-ups were not intended to probe the non-significant three-way interaction but rather to characterize the pattern underlying the significant domain-level Time × Group effect. Pairwise comparisons were Bonferroni-corrected.

**Table 4:** Descriptive statistics of fine motor subscales at T0, T1, and T2 for experimental and control groups.

Fine motor subscale	Group	T0	T1	T2
Eye–hand coordination	Experimental	3.32 (1.25)	3.68 (1.00)	4.11 (0.74)**
	Control	3.20 (1.32)	3.27 (1.28)	3.67 (1.11)
Lateralization	Experimental	4.53 (0.70)	4.53 (0.70)	4.63 (0.60)
	Control	3.00 (1.20)	3.07 (1.16)	3.53 (1.19)
Approach movement	Experimental	3.26 (1.20)	3.74 (1.24)*	3.95 (1.18)*
	Control	3.20 (1.15)	3.27 (1.16)	3.73 (0.96)
Touch	Experimental	3.47 (1.31)	3.68 (1.11)	3.89 (1.15)*
	Control	3.07 (1.16)	3.13 (1.19)	3.47 (1.25)
Grasping	Experimental	2.47 (0.91)	2.79 (0.79)*	2.95 (0.85)*
	Control	2.40 (1.45)	2.40 (1.45)	2.53 (1.46)
Release movement	Experimental	1.58 (1.17)	2.74 (1.45)**	3.26 (1.49)**
	Control	2.20 (1.42)	2.27 (1.39)	1.80 (0.78)
Placement movement	Experimental	1.74 (0.87)	2.16 (0.96)**	2.42 (0.90)**
	Control	1.47 (0.64)	1.47 (0.64)	2.00 (1.25)
Bimanual coordination	Experimental	1.84 (1.01)	2.42 (1.35)**	2.74 (1.37)**
	Control	2.07 (1.03)	2.07 (1.03)	2.07 (1.03)
Pushing or pulling	Experimental	2.11 (1.49)	2.63 (1.21)**	2.95 (1.08)**
	Control	2.33 (1.45)	2.40 (1.40)	2.47 (1.46)
Mean composite score for fine motor skills	Experimental	2.49 (0.88)	3.06 (0.74)**	3.26 (0.80)**
	Control	2.61 (1.13)	2.65 (1.10)	2.89 (1.06)

Values are means with standard deviations in parentheses. The experimental group (*n* = 19) and control group (*n* = 15) were assessed at three time points: T0 (baseline), T1, and T2. *indicate significant difference from T0: *p < 0.05; **p < 0.01.

Supporting Hypothesis 2 (H2), the experimental group demonstrated significant improvements in several fine motor subskills. Eye–hand coordination increased from 3.32 (*SD* = 1.25) at T0 to 4.11 (*SD* = 1.25) at T2 (*p* < 0.01, *d* = 0.75), with minimal changes in the control group. Improvements were also found in approach movement (3.26 (*SD* = 1.20) → 3.95 (*SD* = 1.18); *p* < 0.05, *d* = 0.60), grasping (2.47 (*SD* = 0.91) → 2.95 (*SD* = 0.85); *p* < 0.05, *d* = 0.65), and touch (3.47 (*SD* = 1.31) → 3.89 (*SD* = 1.15); *p* < 0.05, *d* = 0.45). The release movement of the experimental group showed a significant increase at T1 (T0 = 1.58 → T1 = 2.74, *p* < 0.05, *d* = 0.78) and further increased to 3.26 at T2 (*p* < 0.01, *d* = 1.02), indicating progressive skill improvement across sessions. Similar gains were observed in placement movement (1.74 (*SD* = 0.87) → 2.42 (*SD* = 1.35); *p* < 0.01, *d* = 1.00). Bimanual coordination (1.84 → 2.74, *p* < 0.01, *d* = 1.20) and pushing/pulling skills (2.11 (*SD* = 1.02) → 2.95 (*SD* = 1.23); *p* < 0.01, *d* = 1.05) also improved significantly in the experimental group, whereas lateralization remained unchanged in both groups. The mean composite score for fine motor skills rose from 2.49 (SD = 0.88) at T0 to 3.06 (SD = 0.74) at T1 (*p* < 0.05, *d* = 0.64) and 3.26 (SD = 0.80) at T2 (*p* < 0.01, *d* = 0.95) in the experimental group, while the control group’s improvement from 2.61 (SD = 1.13) to 2.89 (SD = 1.06) was not significant. These results confirm H2 and further support H3, highlighting sustained progress across assessment points.

### 3.3 Gross motor functions

Table 5 presents the results for gross motor skills. The ANOVA revealed a significant main effect of Time, *F*(2, 116) = 13.21, *p* < 0.001, *η*^2^ = 0.19, and of Function, *F*(6, 348) = 3.96, *p* = 0.001, *η*^2^ = 0.06, indicating overall improvement and variability across motor tasks. The main effect of Group was not significant, *F*(1, 58) = 1.94, *p* = 0.17, *η*^2^ = 0.03. A significant Time × Group interaction emerged, *F*(2, 116) = 5.02, *p* = 0.008, *η*^2^ = 0.08, showing that the Experimental group improved more markedly than the Control group over time. The Time × Function interaction was significant, *F*(12, 696) = 2.43, *p* = 0.004, *η*^2^ = 0.04, while both the Group × Function, *F*(6, 348) = 1.11, *p* = 0.35, *η*^2^ = 0.02, and the three-way Time × Group × Function interaction, *F*(12, 696) = 1.36, *p* = 0.18, *η*^2^ = 0.02, were not significant. The experimental group showed greater improvements across multiple motor domains, particularly in upright posture, supine-to-prone rolling, supine-to-sitting, sitting-to-standing on a chair, standing-to-sitting on a chair, walking, and spatial orientation (*p* < 0.05 for all; *d* = 0.67, 0.55, 0.60, 0.62, 0.58, 0.59, 0.61, respectively). While the control group started at slightly higher baseline levels in some domains, it showed limited or no improvement over time. More demanding motor tasks (e.g., running, jumping, playing with a ball) remained stable in both groups, suggesting potential floor or ceiling effects. Specifically, baseline scores of running and jumping were close to the minimum scale value (experimental group: running T0 = 1.05 (SD = 0.23), jumping T0 = 1.00 (SD = 0) and remained unchanged at T2), indicating clear floor effects. The difficulty of these tasks exceeded the current abilities of participants (e.g., jumping requires lower limb explosive strength, generally insufficient in RTT patients), so no intervention effect was observed. The mean composite score for gross motor skills in the experimental group increased from 2.50 (SD = 0.49) at T0 to 2.99 (SD = 0.48) at T2 (*p* < 0.05, *d* = 0.87), whereas the control group’s increase from 2.59 (SD = 0.87) to 2.81 (SD = 0.83) did not reach significance. These findings support H2 and H3, demonstrating that gains in gross motor function were both significant and sustained.

**Table 5:** Descriptive statistics of gross motor subscales at T0, T1, and T2 for experimental and control groups.

Gross motor skill	Group	T0 Mean (SD)	T1 Mean (SD)	T2 Mean (SD)
Upright posture	Experimental	3.53 (1.22)	4.05 (1.00)	4.53 (0.61)*
	Control	3.93 (1.33)	4.27 (1.10)	4.53 (0.83)
Sitting posture	Experimental	4.63 (0.50)	4.84 (0.38)	4.95 (0.23)
	Control	3.87 (1.46)	4.07 (1.17)	4.20 (0.86)
Parachute reactions	Experimental	4.58 (0.84)	4.89 (0.32)	5.00 (0.00)
	Control	4.27 (1.28)	3.99 (1.08)	4.10 (0.76)
Rolling: supine to side	Experimental	3.79 (0.85)	4.00 (0.73)	4.32 (0.58)
	Control	3.93 (0.88)	3.87 (0.96)	3.93 (0.90)
Rolling: supine to prone	Experimental	3.11 (1.13)	3.42 (0.96)	3.74 (0.66)*
	Control	2.93 (0.88)	3.00 (0.76)	3.33 (0.62)
Supine to sitting on floor	Experimental	3.07 (0.90)	3.58 (0.91)	3.84 (0.83)*
	Control	2.80 (1.08)	2.93 (1.03)	3.13 (1.13)
Sitting to standing on floor	Experimental	2.26 (0.56)	2.42 (0.61)	2.63 (0.50)
	Control	2.40 (0.83)	2.47 (0.83)	2.60 (0.74)
Sitting to standing on chair	Experimental	3.00 (0.75)	3.42 (1.02)	3.63 (0.96)*
	Control	2.73 (0.96)	2.60 (0.91)	2.73 (0.96)
Standing to sitting on floor	Experimental	2.42 (0.61)	2.47 (0.61)	2.58 (0.77)
	Control	2.33 (0.90)	2.47 (0.99)	2.47 (0.99)
Standing to sitting on chair	Experimental	2.65 (0.62)	3.11 (0.81)	3.37 (0.90)*
	Control	2.67 (1.05)	2.73 (0.96)	2.93 (1.16)
Walking	Experimental	3.74 (1.05)	4.21 (1.03)	4.26 (0.99)*
	Control	3.93 (1.33)	3.93 (1.33)	3.93 (1.33)
Spatial orientation (standing)	Experimental	3.00 (1.53)	3.63 (1.16)	3.84 (1.12)*
	Control	2.80 (1.21)	2.83 (0.99)	2.80 (1.21)
Obstacle crossing	Experimental	2.32 (1.00)	2.95 (1.13)	3.11 (1.10)
	Control	2.80 (1.01)	2.93 (1.03)	3.07 (1.10)
Running	Experimental	1.05 (0.23)	1.05 (0.23)	1.05 (0.23)
	Control	1.60 (0.63)	1.60 (0.63)	1.60 (0.63)
Stairs up and down	Experimental	2.21 (0.79)	2.63 (0.68)	2.68 (0.67)
	Control	2.53 (0.83)	2.73 (0.80)	2.73 (0.80)
Jumping	Experimental	1.00 (0.00)	1.00 (0.00)	1.00 (0.00)
	Control	1.13 (0.35)	1.13 (0.35)	1.13 (0.35)
Picking up an object	Experimental	1.00 (0.00)	1.11 (0.46)	1.26 (0.81)
	Control	1.27 (0.59)	1.27 (0.59)	1.27 (0.59)
Playing with ball	Experimental	1.37 (0.60)	1.35 (0.45)	1.37 (0.49)
	Control	1.87 (0.64)	1.87 (0.64)	1.87 (0.64)
Inclined surface walking	Experimental	3.05 (1.03)	3.32 (0.82)	3.53 (0.77)
	Control	2.67 (1.05)	2.67 (1.05)	2.80 (1.08)
Mean composite score for fine motor skills	Experimental	2.50 (0.49)	2.90 (0.48)	2.99 (0.48)*
	Control	2.59 (0.87)	2.75 (0.85)	2.81 (0.83)

Values are means with standard deviations in parentheses. The experimental group (*n* = 19) and control group (*n* = 15) were assessed at three time points: T0 (baseline), T1, and T2. *indicate significant difference from T0 (*p* < 0.05).

Finally, exploratory correlational analyses within the experimental group revealed moderate-to-strong positive correlations (*r* = 0.46–0.63, all *p* < 0.05) between improvements in composite scores of cognitive and motor skills, suggesting functional integration between these domains. Exploratory analyses stratified by age and mutation type were planned to examine whether these associations were consistent across subgroups. However, due to the limited sample size and absence of individual-level data in each subgroup, these analyses could not be reliably performed. Therefore, the reported correlation refers to the entire experimental group.

## 4 Discussion

The present study investigated the efficacy of an integrated neurorehabilitation program targeting both cognitive and motor domains in individuals with Rett Syndrome (RTT). Consistent with our hypotheses, participants in the experimental group exhibited significant and sustained improvements in neuropsychological and motor functions, whereas those in the control group, engaged in standard educational activities, showed minimal change. Notably, improvements in cognitive and motor domains were positively correlated, supporting the hypothesis of functional integration between these systems.

These findings reinforce the growing body of evidence emphasizing the interdependence of cognitive and motor functions in neurodevelopmental disorders (Di Palma et al., 2025; Fabio et al., 2025a). The observed synergy aligns with theoretical models positing that shared neural substrates—particularly involving the prefrontal cortex, basal ganglia, and cerebellum—underlie both cognitive processing and motor control (Rajda et al., 2025; Hou et al., 2024). The significant improvement in sustained attention in the experimental group (12-s increase from T0 to T2) may be related to the intervention enhancing the activation of the dorsolateral prefrontal cortex (DLPFC), while the improvement in hand motor skills (e.g., grasping, releasing) may be associated with enhanced synaptic plasticity in the cerebellar vermis. This is consistent with Di Palma et al.’s (2025) conclusion that “cognitive-motor interventions improve function by regulating the prefrontal-cerebellar pathway,” further verifying the intervention’s targeted effect on key neural networks in RTT patients. Our results extend this framework to the RTT population, showing that cognitive-motor training can yield measurable and clinically relevant benefits even in individuals with severe and complex impairments.

The significant improvements in attention, memory, and temporal sequencing support previous work demonstrating that neuropsychological functions are modifiable through targeted interventions, even in conditions traditionally considered static or degenerative (Gangemi et al., 2018; Fabio et al., 2021). Improvements in sustained attention and problem-solving, in particular, mirror findings from studies on ADHD and ASD, where cognitively engaging motor interventions have produced medium to large gains in executive functions (Fang et al., 2024; Zhao et al., 2024; Wang et al., 2015). Compared with Fabio et al.’s (2021) eye-tracking-based cognitive rehabilitation study, the improvement in neuropsychological skills observed in this study (e.g., a 12-s increase in sustained attention) is greater than the 8-s increase reported in the former, which may be attributed to the synergistic enhancement of cognitive performance by the added motor training. Similarly, compared with Fonzo et al.’s (2020) motor-only rehabilitation study, the improvement in hand motor skills in this study (e.g., 1.68-point increase in release movement at T2) surpasses the 0.9-point increase observed previously, highlighting the advantages of integrated cognitive-motor interventions and demonstrating the incremental contribution of this study to RTT rehabilitation research.

Parallel gains in gross and fine motor skills further underscore the efficacy of the integrated approach. These improvements are in line with previous studies highlighting the importance of individualized physiotherapy in maintaining and enhancing motor function in RTT (Fabio et al., 2020; Fabio et al., 2025a; Romano et al., 2020). However, our findings go beyond prior research by demonstrating that motor improvements can be amplified when coupled with cognitive stimulation, possibly due to enhanced engagement, neuroplasticity, or attentional regulation during training (Li et al., 2025; Gao et al., 2024).

Importantly, the presence of cumulative effects over the two treatment cycles suggests that the benefits of integrated training are not transient but build over time. This temporal dynamic supports the need for sustained, longitudinal interventions and aligns with research in TBI and developmental coordination disorder advocating for continuous, multidimensional rehabilitation (Shen et al., 2025; Gao et al., 2024).

The positive association between cognitive and motor improvements should be interpreted cautiously. Because the intervention simultaneously targeted both domains, parallel gains may reflect concurrent exposure rather than genuine cross-domain transfer or functional integration. A design including separate motor-only and cognitive-only training groups would be required to test whether improvements in one domain can causally promote gains in the other. This interrelation mirrors neuroimaging evidence showing overlapping activation in frontoparietal and cerebellar circuits during both motor and cognitive tasks (Good et al., 2021; Di Palma et al., 2025).

### 4.1 Limitations

Despite these promising findings, several limitations should be acknowledged. First, the sample size was relatively small, reflecting the rarity of RTT and the intensive nature of the intervention. This study has a small sample size (*n* = 34). In the future, sample size can be expanded to over 100 participants through multi-center collaboration (e.g., joining 3–5 RTT specialized rehabilitation centers), and different RTT subtypes (e.g., classic RTT, atypical RTT) can be included to improve representativeness. Meanwhile, Bootstrap resampling (1,000 repetitions) can be used in small-sample analyses to verify the robustness of correlation results and reduce the impact of sampling error on conclusions.

While the statistical power was sufficient to detect medium to large effects, replication in larger samples is necessary to confirm generalizability. Second, randomization occurred within pre-existing rehabilitative settings, which might have introduced contextual or environmental biases, despite baseline comparability. Third, the control group did not receive a placebo or alternative active intervention, raising the possibility that observed effects were partially due to increased therapeutic attention or expectancy effects.

In addition, the measures used, although ecologically valid and clinically informative (e.g., GAIRS subscales), were based primarily on behavioral observations rather than objective neurophysiological markers. Future studies could complement behavioral observations with performance-based measures and neurophysiological assessments (e.g., EEG or fMRI) to obtain more objective and sensitive indicators of cognitive and motor improvements.

### 4.2 Clinical and theoretical implications

Despite these limitations, the current findings have important theoretical and clinical implications. From a theoretical standpoint, they contribute to the growing evidence that cognitive and motor functions are dynamically linked and should be conceptualized as part of an integrated functional system. From a clinical perspective, the study supports the implementation of multimodal, interdisciplinary interventions in RTT and potentially other neurodevelopmental disorders. In clinical practice, the intervention protocol can be integrated into school curricula (e.g., three after-school rehabilitation sessions per week, led by trained school therapists). Meanwhile, families can be provided with ‘simple training kits’ containing tools such as colored rings and illustrated story cards, along with video guidance to facilitate home-based auxiliary training (e.g., 15 min of daily fine motor practice). Regular therapist-family communication meetings (every 2 months) are recommended to adjust training difficulty and ensure alignment between school and home interventions, supporting maintenance of long-term effects. Such protocols may not only enhance specific skills but also promote broader developmental outcomes by leveraging the interaction between cognitive engagement and motor practice.

In conclusion, the integrated cognitive-motor training implemented in this study proved to be an effective strategy for improving both neuropsychological and motor abilities in individuals with RTT. These results highlight the importance of addressing the interconnected nature of functional domains in neurorehabilitation and advocate for the adoption of synchronized, multidisciplinary approaches in clinical practice.

## Data Availability

The datasets generated and analyzed during the current study are available in the Open Science Framework (OSF) repository, at: https://osf.io/2563x/overview?view_only=6f8500ce6edd4df584bab7713cdef165.
