# Supplementary material for: Enhancing cognitive-motor recovery in Rett syndrome: effects of integrated intervention on neuropsychological and motor outcome
Source: Front Psychol. 2025 Nov 5;16:1679593. doi: 10.3389/fpsyg.2025.1679593 (PMC12626818; doi:10.3389/fpsyg.2025.1679593)
Supplement: Supplementary file 1 [file Supplementary_file_1.docx]

**Appendix A**

Description of the assessment procedure for each item of the GAIRS.

| Ability | Assessment Procedure |  |
| --- | --- | --- |
| **Neuropsychological Concepts** | |  |
| Temporal orientation | This ability is evaluated with the collaboration of the caregivers of the participant as it tests the ability to understand temporal orientation in daily activities (e.g., if the participant understands when the mother says, “First finish your meal and then you can watch TV” and asks the participant “If you finish your meal what can you do? Please look at it”). |  |
| Spatial orientation | This ability is evaluated with the collaboration of the caregivers of the participant as it tests the ability to understand spatial concepts in daily activities (e.g., if the participant understands when the mother says “look at the ball in the box”). |  |
| Memory span | The participant sits at a table in front of the therapist. The therapist places five objects or images representing different common objects in every participant's lap or on a table in front of her. The objects/images are placed 40 cm from each other. In this way all the objects/images are observable for 1 minute and within easy reach and grasp of the participants; then the therapist removes the objects/images and places two objects/images, one just presented and one as a distractor; location of the objects/images on the right or on the left takes place in a random order. The therapist then asks the participant “Have you seen this object/image before?”. The same probe must be used 3 times. |  |
| Logical sequencing: | The participant sits at a table in front of the therapist. The therapist presents the participant with a logical sequence, for example “a participant throws a ball, so the window breaks”, then asks the participant to recognize the cause (throwing the ball) and effect (breaking the window) represented on two different images with two distractors. The same probe must be used 5 times. |  |
| Categorization | The participant sits at a table in front of the therapist. The therapist presents the participant with items of a specific category (e.g., animals or food) with some distractors and asks the participants to recognize items of the category, for example: “Look at the dog. What is this? Or this other?” showing two cards with animals and flowers. The same probe must be used 10 times. |  |
| Temporal concepts | The participant is sitting at a table and has three images of a time sequence in the correct order on the table; the therapist explains and indicates the right sequence and then positions two images, one on the left and one on the right. The participant is asked to look at them and to choose the target (e.g., before) by looking at it and touching it (eye-hand coordination). If the participant does not have the motor ability to touch, only the eye response is considered. The position of the stimuli is changed, and the whole procedure is repeated three times with the stimuli in random order (right left and vice-versa). This procedure is repeated for each target of the category. The therapist presents all the target stimuli (3), one at a time, and registers all the responses of the participants: for each target, only if the participant performs all 3 correct answers (on the right and left positions) is the target noted as already acquired. |  |
| **Hand motor skills** | |  |
| Eye–hand coordination | The participant sits at the table with a motivating object placed in her hand. The participant must look at the object she holds in her hand. The therapist asks her to look at the object in her hand 5 times, varying the objects. |  |
| Lateralization | The participant sits at the table with a motivating object to reach or grasp. The object is moved to a different point on the table, and the therapist tells her “Please reach for it”, for 5 times. |  |
| Approach movement | The participant sits at the table. The object to be reached is placed on the table, on the midline, and at a distance corresponding to half the length of the participant’s arm. The dominant hand is held on the table at the bottom edge; the non-dominant hand is gently kept blocked under the table. After the object is shown enthusiastically to the participant, the examiner says, “please reach for it”. This procedure is carried out 5 times; the time allowed for the participant to activate her arm is 10 seconds, after which the request is interrupted. |  |
| Touch | The participant sits at the table. The object to be taken is placed on the table, on the midline. The dominant hand is held on the table at the bottom edge; the non-dominant hand is gently kept blocked under the table. After the object is shown enthusiastically to the participant, the examiner says, “touch it”. This procedure is carried out 4 times: firstly, the object is placed at less than 5cm, then between 5 and 10 cm, then at more than 10 cm and finally in a different position. The time allowed for the participant to activate her arm is 10 seconds, after which the request is interrupted. |  |
| Grasping | The participant sits at the table. The object to be taken is placed on the table, on the midline. The dominant hand is held on the table at the bottom edge; the non-dominant hand is gently kept blocked under the table. After the object is enthusiastically shown to the participant, the examiner says, “grasp it”. This procedure is carried out 4 times: two times with a small object and two times with a bigger object. The time allowed for the participant to activate her arm is 10 seconds, after which the request is interrupted |  |
| Release movement | The participant sits, and the non-dominant hand is gently kept blocked; below the chair there is a big basket. After the object is enthusiastically shown to the participant, the therapist puts it in the palm participant’s hand and says, “release it”. This procedure is carried out 4 times: first, the ball has to be put into a big basket, then into a box, then onto a dish and finally onto the partner's hand. The time allowed for the participant to activate her arm is 10 seconds, after which the request is interrupted. |  |
| Placement movement | The participant sits at the table. A tower of two cubes is placed on the table, on the midline. The dominant hand is held on the table at the bottom edge; the non-dominant hand is kept gently blocked under the table. After the object is enthusiastically shown to the participant, the therapist says, “please remove or replace one block”. This procedure is carried out 3 times: the first time the participant has to remove it, the second she has to reposition it and the third she has to replace it. The time allowed to the participant to activate her arm is 10 seconds, after which the request is interrupted. |  |
| Bimanual coordination | The therapist is near the participant, who is seated at a table.  The therapist places in front of the participant a motivating object or activity that requires the use of both hands (e.g., pulling apart Velcro, opening a container, threading a large bead). The therapist says to the participant “please, do it”. This procedure is carried out 4 times, with a gradual reduction in assistance and with increasing complexity of the task (e.g., greater precision, strength, or coordination required). |  |
| Pushing or pulling | The participant sits at the table. A toy is placed on the table, on the midline. The dominant hand is held on the table at the bottom edge; the non-dominant hand is gently kept blocked under the table. The therapist says, “please pull and push it”. This procedure is carried out 3 times: first with the object at 1 or 2 cm, then at a distance between 3 and 10 cm and finally at a distance more than 10cm. The time allowed for the participant to activate her arms is 10 seconds, after which the request is interrupted. |  |
| **Global motor abilities** | |  |
| Static balance | The therapist stands in front of the participant and the participant is positioned with the help of the therapist. The therapist says to her “please, stay in this posture alone”. This procedure is carried out 4 times with a gradual reduction in aid and a gradual increase in posture maintenance time. |  |
| Sitting posture | The therapist stands in front of the participant, and the participant sits on the floor. The therapist tells her, “Please, sit down”. This procedure is carried out 3 times and the therapist takes note of how the participant is sitting. |  |
| Parachute reactions | The therapist stands in front of the participant, and the participant sits on the floor. The therapist applies light thrusts at the level of the shoulders; the participant must put out her hands to prevent a fall. This procedure is carried out 3 times: the thrusts are front, side and, finally, from behind. |  |
| Rolling: supine to side | The therapist sits near the participant. The participant lies on her back. The therapist says to the participant “please, roll onto one side”. This procedure is carried out 4 times with a gradual reduction in aid. |  |
| Rolling: supine to prone | The therapist is sitting near the participant. The participant is lying on her back.  The therapist tells the participant to roll. This procedure is carried out 4 times with a gradual reduction  in aid. |  |
| Supine to sitting on floor | The therapist is sitting near the participant. The participant is lying on her back.  The therapist says to the participant “please, sit on the floor”. This procedure is carried out 4 times  with a gradual reduction in aid. |  |
| Sitting standing on floor | The therapist is sitting near the participant. The participant is sitting on the floor.  The therapist says to the participant “please, stand up”. This procedure is carried out 4 times with a gradual reduction in aid. |  |
| Sitting to standing on chair | The therapist is standing in front of the participant. The participant is sitting on the Chair.  The therapist says to the participant “please, stand up”. This procedure is carried out 4  times with a gradual reduction in aid. |  |
| Standing sitting on floor | The therapist is standing in front of the participant. The participant is standing in  front of the therapist. The therapist says to the participant “please, sit down on the floor”. This  procedure is carried out 4 times with a gradual reduction in aid. |  |
| Standing sitting on chair | The therapist is standing in front of the participant. The participant is standing in  front of the therapist; the chair is behind the participant. The therapist says to the participant “please, sit down on the chair”. This procedure is carried out 4 times with a gradual reduction in aid. |  |
| Walking | The therapist is in front of the participant who is standing, the therapist places a  motivating object on the other side of the room. The therapist says to the participant “please, go to  the object”. This procedure is carried out 4 times with a gradual reduction in aid and a gradual increase in the number of steps. |  |
| Spatial orientation (standing) | The therapist is in front of the participant who is standing; the therapist says to the  participant “please, move” in relation to follow and gestural direction. This procedure is carried out  3 times with different instructions: one time the participant has to turn, one time she has to rotate, one time she has to change direction behind her |  |
| Obstacle crossing | The therapist is in front of the participant who is sitting with support behind her; the therapist says to the participant “play with the ball” and he tries to throw or receive the ball. This  procedure is carried out 4 times with a gradual reduction in aid. |  |
| Running | The therapist is in front of the participant who is standing, the therapist places a  motivating object on the other side of the room. The therapist says to the participant “run to the  object”. This procedure is carried out 4 times with a gradual reduction in aid. |  |
| Stairs up and down: | The therapist near the participant who is standing in front of the staircase; the  therapist places a motivating object at the end of the stairs. The therapist says to the participant “climb  up/descend stairs”. This procedure is carried out 4 times with a gradual reduction in aid. |  |
| Jumping | The participant stands in front of the therapist. The therapist put a motivating object on the  floor, and he says, “pick up the object from the ground”. This procedure is carried out 4 times with  a gradual reduction in aid. |  |
| Dynamic Balance | The therapist is near the participant, who is standing upright in the middle of the room; the therapist places a motivating object a few meters away in a direction that requires the participant to change position or direction while moving. He then says to the participant “please, go”. This procedure is carried out 4 times, with a gradual reduction in physical or verbal assistance and with an increasing complexity of the path (e.g., including turns, changes of direction, or obstacles). |  |
| Playing with ball: | The therapist is in front of the participant who is sitting with a support behind her. the therapist says to the participant “play with the ball” and he tries to throw or receive the ball. This procedure is carried out 4 times with a gradual reduction in aid. |  |
| Inclined surface walking: | The therapist is near the participant who is standing at the beginning of the slope; the therapist places a motivating object at the end of the slope, and he says to the participant “please, go”. This procedure is carried out 4 times with a gradual reduction in aid and with an increase in the number of steps required |  |
|  |  |  |

**Appendix B**

Detailed summary of the intervention protocol, including task examples, time allocation, and progression criteria.

| **Phase** | **Duration** | **Activities** | **Materials** | **Progression Criteria** |
| --- | --- | --- | --- | --- |
| Warm-up | 5–10 min | Interactive songs with gestures, visual cues, assisted arm movements | Illustrated cards, simple songs, visual cues | Increase complexity of gestures (from single to combined sequences) |
| Cognitive / Neuropsychological tasks | 20–30 min | - Temporal orientation (today/tomorrow; short illustrated story sequencing)  - Spatial orientation (above/below; object placement tasks)  - Memory span (recall 2–3 objects, gradually up to 5–6)  - Categorization (grouping by color, shape, or size)  - Logical sequencing (ordering 3–6 pictures) | Picture cards, story sequences, small object sets (toys, shapes) | Gradual increase in number of items (e.g., from 3 to 6), complexity of categories (single → multiple features) |
| Fine motor training | 10–15 min | Grasping and release - Eye–hand coordination - Bimanual tasks (e.g., stacking, inserting objects) | Stacking rings (diameter 3–5 cm; start with 5 rings → up to 10), cups, containers | Progression in number of items, reduction of support |
| Gross motor training | 10–15 min | - Sitting posture, static balance - Rolling (supine → side → prone)  - Obstacle crossing and assisted walking  – Balance exercises (supported sitting → unsupported sitting → standing)  - Ball play (throwing, catching, kicking) | Therapy mat, soft obstacles, small ball, Fit ball | Progression from assisted to semi-independent movements; increasing complexity |
| Closure / Transition | 2–5 min | Relaxation or simple interaction to end session | Visual symbols, songs | Maintain predictability to support generalization |
